# Altered functional connectivity of amygdala underlying the neuromechanism of migraine pathogenesis

**DOI:** 10.1186/s10194-017-0722-5

**Published:** 2017-01-23

**Authors:** Zhiye Chen, Xiaoyan Chen, Mengqi Liu, Zhao Dong, Lin Ma, Shengyuan Yu

**Affiliations:** 1 Department of Radiology, Chinese PLA General Hospital, 28 Fuxing Road, Beijing, 100853 China; 2 Department of Neurology, Chinese PLA General Hospital, 28 Fuxing Road, Beijing, 100853 China; 3 Department of Radiology, Hainan Branch of Chinese PLA General Hospital, Beijing, 100853 China

**Keywords:** Migraine, Amygdala, Neurolimbic pain network, Functional connectivity, fMRI

## Abstract

**Background:**

The amygdala is a large grey matter complex in the limbic system, and it may contribute in the neurolimbic pain network in migraine. However, the detailed neuromechanism remained to be elucidated. The objective of this study is to investigate the amygdala structural and functional changes in migraine and to elucidate the mechanism of neurolimbic pain-modulating in the migraine pathogenesis.

**Methods:**

Conventional MRI, 3D structure images and resting state functional MRI were performed in 18 normal controls (NC), 18 patients with episodic migraine (EM), and 16 patients with chronic migraine (CM). The amygdala volume was measured using FreeSurfer software and the functional connectivity (FC) of bilateral amygdala was computed over the whole brain. Analysis of covariance was performed on the individual FC maps among groups.

**Results:**

The increased FC of left amygdala was observed in EM compared with NC, and the decreased of right amygdala was revealed in CM compared with NC. The increased FC of bilateral amygdala was observed in CM compared with EM. The correlation analysis showed a negative correlation between the score of sleep quality (0, normal; 1, mild sleep disturbance; 2, moderate sleep disturbance; 3, serious sleep disturbance) and the increased FC strength of left amygdala in EM compared with NC, and a positive correlation between the score of sleep quality and the increased FC strength of left amygdala in CM compared with EM, and other clinical variables showed no significant correlation with altered FC of amygdala.

**Conclusions:**

The altered functional connectivity of amygdala demonstrated that neurolimbic pain network contribute in the EM pathogenesis and CM chronicization.

## Background

Migraine is a common type of primary headaches with a reported prevalence of about 5.7% in men and 17.0% in women [1], and the prevalence of migraine is 9.3 of general population [2] in China. Migraine is also a major cause of chronic headaches, approximately 2.5% of episodic migraine (EM) is transformed to chronic migraine (CM) [3]. Although the growing studies supported the key roles of cortical spreading depression (CSD) and trigeminovascular system in the migraine pathogenesis, the rigorous neuromechanism of EM and the road to migraine chronicization were not completely understood and became to be worth paying close attention.

Beyond neurovascular model, a dysfunctional neurolimbic pain network model expands the conventional concept about migraine, and it could help understand the migraine attack, the migraine chronicization and refractoriness [4]. Recently, increased connectivity between amygdala and visceroceptive cortex in migraine was observed, and which confirmed the roles of neurolimbic pain network dysfunction in the migraine pain genesis [5]. The central sensitization of migraine was confirmed in the animal model by Malick et al. in 2000 [6], and this model could account for many of the temporal and sysmptomatic features of migraine [7]. Ongoing researches demonstrated that central sensitization could reach critical clinical implications for chronic head and neck pain [8]. Therefore, we propose that neurolimbic pain network may bridge the gap between the neurovascular model and central sensitization model.

Based on the current knowledge, the brain regions related with pain processing and modulation mainly included prefrontal cortex, basal ganglia, thalamus, cingulate cortex, insular, cerebellum and periaqueductal gray matter (PAG). Some studies demonstrated that amygdala also involved the migraine pain modulation, such as the modulation of synaptic transmission by CSD [9] and the PAG network [10] and chronic migraine. The amygdala is a large grey matter complex in the limbic system, and it plays a key role in emotion, motivation, learning and memory, and also in migraine [11].

Therefore, the motivation of this study is to investigate the amygdala structural and functional changes in migraine and to elucidate the mechanism of neurolimbic pain-modulating in the migraine pathogenesis. We hypothesized that amygdala was involved in the pain modulation in migraine. To address this hypothesis, we obtained structural and functional MR images for normal controls (NC), episodic migraine (EM) patients, and chronic migraine (CM) patients. Firstly, the amygdala volume was automatically measured based on the structural images using FreeSurfer software, and comparisons of between-group analysis were performed. Secondly, the functional connectivity of bilateral amygdala was computed based on resting-state fMRI, and comparisons of between-group analysis were performed to explore the altered functional connectivity in CM compared with EM.

## Methods

### Subjects

Fifty-two subjects were enrolled, including 18 patients with EM and 16 patients with CM, and 18 NCs with matched age and gender. Patients were recruited from International Headache Center, Department of Neurology, Chinese PLA General Hospital, and inclusion criteria was based on the International Classification of Headache Disorders, third Edition (beta version) (ICHD -3 beta) [12]. All the subjects underwent a standard categorical four-grade sleep disturbance scale (SDS) (0, normal; 1, mild sleep disturbance; 2, moderate sleep disturbance; 3, serious sleep disturbance), Visual Analogue Scale(VAS), Hamilton Anxiety Scale (HAMA), Hamilton Depression Scale (HAMD) and Montreal Cognitive Assessment (MoCA) evaluation. The exclusion criteria were the following: cranium trauma, illness interfering with central nervous system function, psychotic disorder, and regular use of a psychoactive or hormone medication, and onabotulinumtoxin A medication. All the CM patients did not experience medication overuse. NCs were recruited form hospital staffs and their relatives. All the subjects received general physical examination and neurological examination and were normotensive (≤140/90 mmHg), and free from cardiovascular, metabolic and psychiatric disorders. All the subjects were right-handed and underwent MRI conventional examination to exclude the subjects with cerebral infarction, malacia or occupying lesions. The alcohol, nicotine, caffeine and other substances were avoided at least 12 h before MRI examination. Written informed consent was obtained from all participants according to the approval of the ethics committee of the Chinese PLA General Hospital.

### MRI acquisition

MRI scans were taken in the interictal stage at least 3 days after a migraine attack, and no migraine preventive medication was used in the past 3 months. Images were acquired on a GE 3.0T MR system (DISCOVERY MR750, GE Healthcare, Milwaukee, WI, USA) and a conventional eight channel quadrature head coil was used. All the subjects were instructed to lie in a supine position, and form padding was used to limit head movement. Conventional T2-weighted images were obtained first. Then a high resolution three-dimensional T1-weighted fast spoiled gradient recalled echo(3D T1-FSPGR) sequence was performed, which generated 360 contiguous axial slices [TR (repetition time) = 6.3 ms, TE (echo time) = 2.8 ms, flip angle = 15o, FOV (field of view) = 25.6cm × 25.6cm, Matrix = 256 × 256, slice thickness = 1 mm]. Lastly, the resting-state fMRI was performed, where subjects were instructed to relax, keep their eyes closed, stay awake, remain still, and clear their heads of all thoughts. Functional images were obtained by using a gradient echo-planar imaging (EPI) sequence (TR = 2000 ms, TE = 30 ms, flip angle = 90, slice thickness = 4mm, slice gap = 1 mm, FOV = 24cm × 24cm, Matrix = 64 × 64), and 180 continuous EPI functional volumes were acquired axially over 6 min. All the subjects did not complain any discomfort and feel asleep during scanning. No obvious structural damage was observed based on the conventional MR images.

### Data processing

MR structural images were processed using Freesurfer software (version 4.3.0) (http://surfer.nmr.mgh.harvard.edu/fswiki/FreeSurfer), which was run using the Linux 2.6.15-2.5 operating system. The amygdala volume could be automatically measured using automated labeling of neuroanatomical structures technique, and the accuracy has been validated comparing with manual labeling [13]. The preprocessing steps have been elucidated in previous studies [13, 14].

Functional images were processed using Statistical Parametric Mapping 8 (SPM8) (http://www.fil.ion.ucl.ac.uk/spm) and resting-state fMRI data analysis toolkit (REST v1.8) [15] running under MATLAB 7.6 (The Mathworks, Natick, MA, USA).

The data preprocessing was carried out as following: (1) The first ten volumes of each functional time course was discarded to allow for T1 equilibrium and the participants to adapt; (2) Slice timing; (3) Head motion correction; (4) Spatial normalization. These steps were performed by SPM8. No subjects had head motion with more than 1.5mm displacement in X, Y, and Z direction or 1.50 of any angular motion throughout the course of the scanning. The linear trend removal and temporal band-pass filtering (0.01–0.08 Hz) was performed by REST [15].

**Fig. 1:**
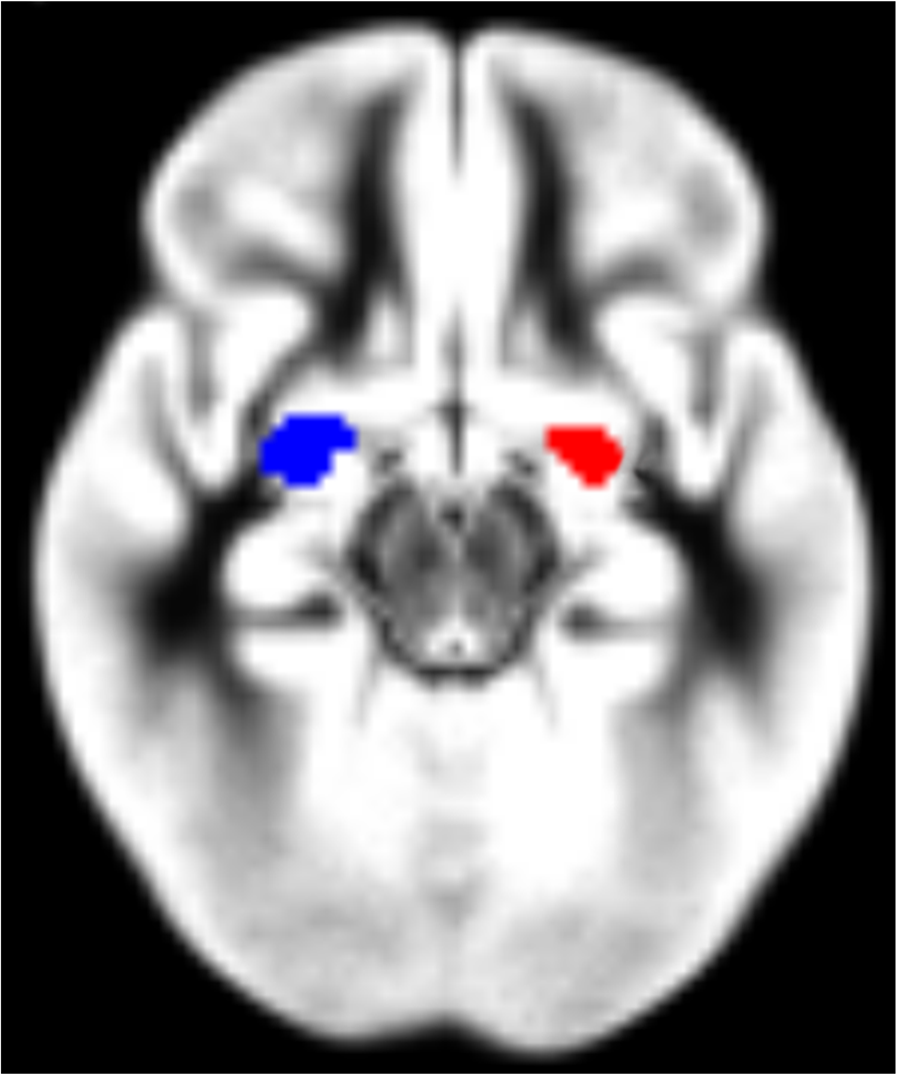
The bilateral amygdala masks were generated based on the AAL template. *Red*, left amygdala; *Blue*, right amygdala

The functional connectivity analysis was performed as following: (1) Spatial smooth (full width at half maximum (FWHM) = 6 mm) using SPM8; (2) Amygdala was defined using AAL template [16] (Fig. 1), then the amygdala was resliced with the brainmask template with 63 × 71 × 63 size to avoid the some voxels outside the brain; (3) Functional connectivity computation of the left and right amygdala were performed using REST(v1.8). The time course of bilateral amygdala were extracted, and Pearson correlation were used to calculated functional connectivity between the extracted time course and the averaged time courses of the whole brain in a voxel-wise manner. The white matter, CSF, and the six head motion parameters were used as covariates. (4) The individual r-maps were normalized to Z-maps using Fisher’s Z transformation. (5) The positive clusters based on the statistical parametric mapping were generated binary mask, and the connectivity strength of the positive brain region was extracted based on the Z-maps.

### Statistical analysis

The age, education years, VAS, HAMA, HAMD and MoCA were performed with one-way analysis of variance (ANOVA), disease duration (DD) was performed with two-sample *t* test. Post Hoc multiple comparisons were performed by LSD methods with equal variances and Dunnett’s T3 with unequal variances. The amygdala volume was compared using Analysis of covariance (ANCOVA) among each group, covarying for age, gender and education years. The Pearson correlation analysis was performed between disease duration, VAS and amygdala volume. These statistics was processed using IBM SPSS 19.0, and the *P* value of less than 0.05 was considered to indicate a statistically significant difference.

Analysis of covariance (ANCOVA) was performed to identify the regions with significant differences in connectivity to amygdala between groups, covarying for age, gender, education years. Significance was set at a *P* value of < 0.001 without correction. The minimal number of contiguous voxels was set at 10. The statistical maps were masked on SPM8 T1 template.

Bivariate correlation analysis was applied to the connectivity strength of the positive brain region in each compared groups. The Pearson’s method was performed for the scaled variable, and the Spearman’s method was performed for the ordinal variables. The *P* value of less than 0.05 was considered to indicate a statistically significant difference.

## Results

### Demography and neuropsychological test

Demography and neuropsychological scores were shown in Table 1. Age, education years, HAMD score and MoCA score showed no significant difference among each group (*P* > 0.01). HAMA score in NC (9.7 ± 3.2) was lower than that in CM (21.6 ± 11.0), and other groups showed no significant difference among them.

**Table 1:** The clinical characteristics of the subjects

	NC	EM	CM
Num(F/M)	18(14/4)	18(14/4)	16(12/4)
Age	39.1 ± 10.0	33.4 ± 11.0	42.4 ± 8.6
DD	NA	12.4 ± 8.1	11.3 ± 9.3
EduYears	13.7 ± 3.2	13.7 ± 3.7	10.8 ± 3.8
SDS	NA	1.27 ± 1.0	2.4 ± 1.1
VAS	NA	8.3 ± 1.5	7.9 ± 1.5
HAMA	9.7 ± 3.2	15.7 ± 9.85	21.6 ± 11.0
HAMD	15.9 ± 2.9	10.9 ± 7.3	16.3 ± 10.5
MoCA	26.9 ± 2.5	29.2 ± 1.5	22.9 ± 5.4

*DD* disease duration, *EduYears* education years, *SDC* sleep disturbance scale, *VAS* visual analogue scale, *HAMA* Hamilton anxiety scale, *HAMD* Hamilton depression scale, *MoCA* Montreal Cognitive Assessment, *NC* normal control, *EM* episodic migraine, *CM* chronic migraine

### Comparison of amygdala volume among each group

The amygdala volume showed no significant difference among NC (left, 1.61 ± 0.32 ml; right, 1.64 ± 0.25 ml; mean, 1.62 ± 0.27ml), EM(left, 1.59 ± 0.26ml; right, 1.64 ± 0.21 ml; mean, 1.62 ± 0.23 ml) and CM (left, 1.62 ± 0.23 ml; right, 1.68 ± 0.20 ml; mean, 1.65 ± 0.20 ml), although the amygdala volume in CM showed the increased trend compared with that in NC and EM. The correlation analysis demonstrated that there was no significant correlation between VAS, MMSE, HAMA, HAMD, MoCA score and amygdala volume.

### Comparison of functional connectivity of amygdala between NC and EM

**Fig. 2:**
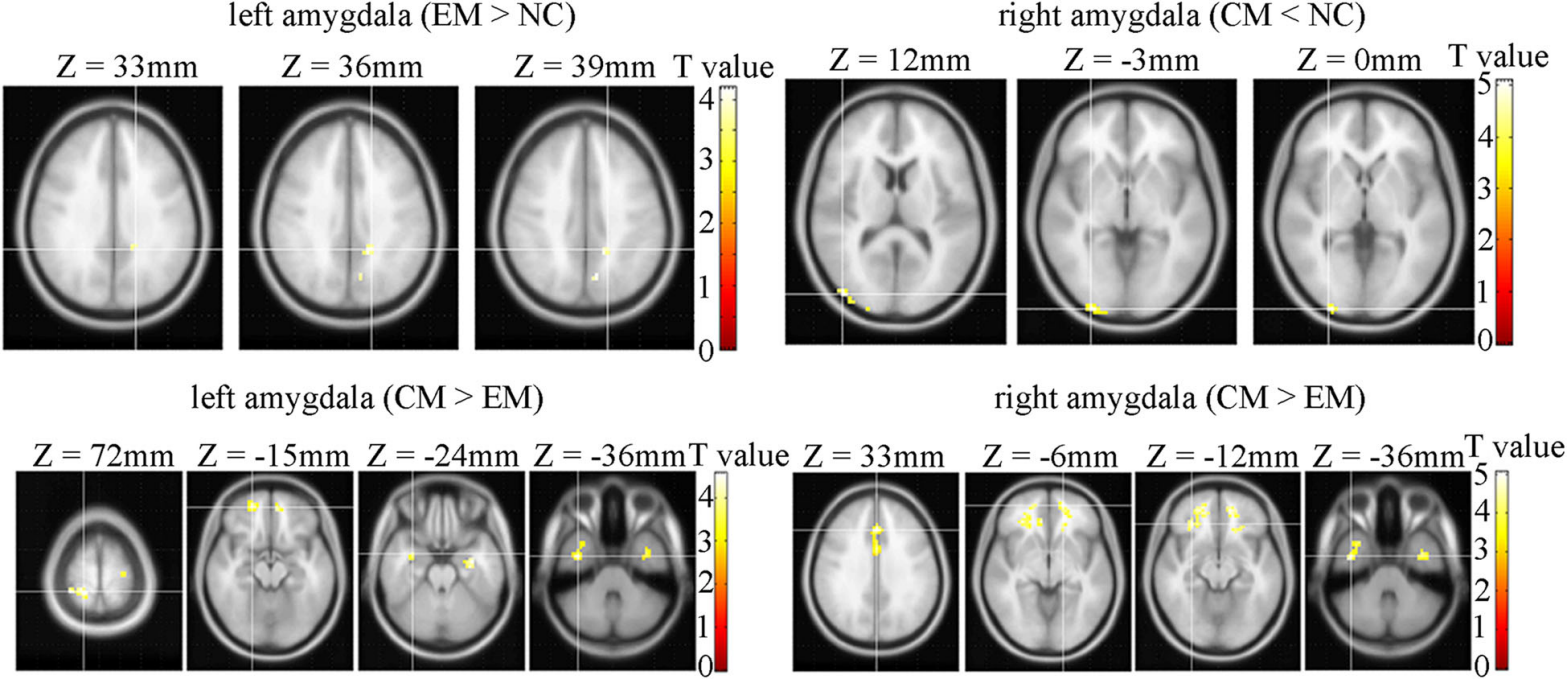
Comparison of FC of amygdala among NC, EM and CM. Warm color represents altered functional connectivity. EM > NC, increased FC of amygdala in EM compared with NC; CM < NC, decreased FC of amygdala in CM compared with NC; CM > EM, increased FC of amygdala in CM compared with EM

It was demonstrated that the brain regions with increased FC of the left amygdala mainly located in the left middle cingulate gyrus ([-18 -45 36], T value 4.18) and left precuneus ([-9 -66 39], T value 4.14) in EM compared with NC (Fig. 2). However, the increased FC of right amygdala could not be revealed in EM compared with NC. The decreased FC of bilateral amygdala was not observed in EM compared with NC.

### Comparison of functional connectivity of amygdala between NC and CM

There were no significant changes for FC of left amygdala between NC and CM. The brain regions with decreased FC of the right amygdala mainly located in right inferior occipital lobe ([30 -99 -3], T value 4.26) and right middle occipital lobe ([45 -83 12], T value 4.11) in CM compared with NC (Fig. 2). There was no increased significant change for FC of the right amygdala in CM compared with NC.

### Comparison of functional connectivity of amygdala between EM and CM

Table 2 and Fig. 2 showed the increased functional connectivity of bilateral amygdala in CM compared with EM. The common brain regions with increased connectivity located in bilateral inferior temporal gyri.

**Table 2:** The brain regions with increased functional connectivity of bilateral amygdala in CM patients compared with EM in resting state

	Anatomic region	MNI-space			K value	*P*_*un*corr_value	T value
		X	Y	Z			
L-Amygdala	Temporal_Inf_L	-33	-6	-42	33	0.000	5.90
	Temporal_Inf_R	39	-3	-33	35	0.000	5.72
	Frontal_Sup_Orb_R	21	51	-15	36	0.000	5.08
	Fusiform_L	-33	-9	-24	28	0.000	4.96
	Postcentral_R	18	-38	72	28	0.000	4.58
	Rectus_L	-6	48	-15	19	0.000	4.53
	Amygdala_R	33	0	-24	10	0.000	4.28
	Precentral_L	-24	-18	75	12	0.000	3.85
R-Amygdala	Temporal_Inf_L	-33	-6	-42	47	0.000	6.49
	Cingulum_Mid_L	0	27	33	113	0.000	5.67
	Temporal_Inf_R	42	-3	-36	45	0.000	4.79
	Frontal_Med_Orb_L	-15	54	-6	68	0.000	4.61
	Temporal_Pole_L	-42	15	-33	11	0.000	4.48
	Frontal_Inf_Orb_R	30	33	-12	106	0.000	4.46
	Cingulum_Ant_R	9	36	-6	10	0.000	4.29
	Frontal_Inf_Orb_L	-24	24	-18	45	0.000	4.19

*MNI* Montreal Neurological Institute, *X, Y, Z* coordinates of the primary maximum of the cluster; (T > 3.39, *P* < 0.001) (without correction)

The difference brain regions with increased connectivity were observed for bilateral amygdala. The remained brain regions with increased connectivity of left amygdala mainly located in the right orbital part of superior frontal gyrus, left fusiform, right postcentral gyrus, left rectus, right amygdala and left precentral gyrus in CM compared with EM. The remained brain regions with increased connectivity of right amygdala mainly located in the left middle cingulate gyrus, left orbital part of medial frontal gyrus, left temporal pole, right orbital part of inferior frontal gyrus, right anterior cingulate gyrus and left orbital part of inferior frontal gyrus. The decreased FC of bilateral amygdala was not observed in CM compared with EM.

### Correlation analysis of the connectivity strength of the positive brain regions with the clinical variables

**Fig. 3:**
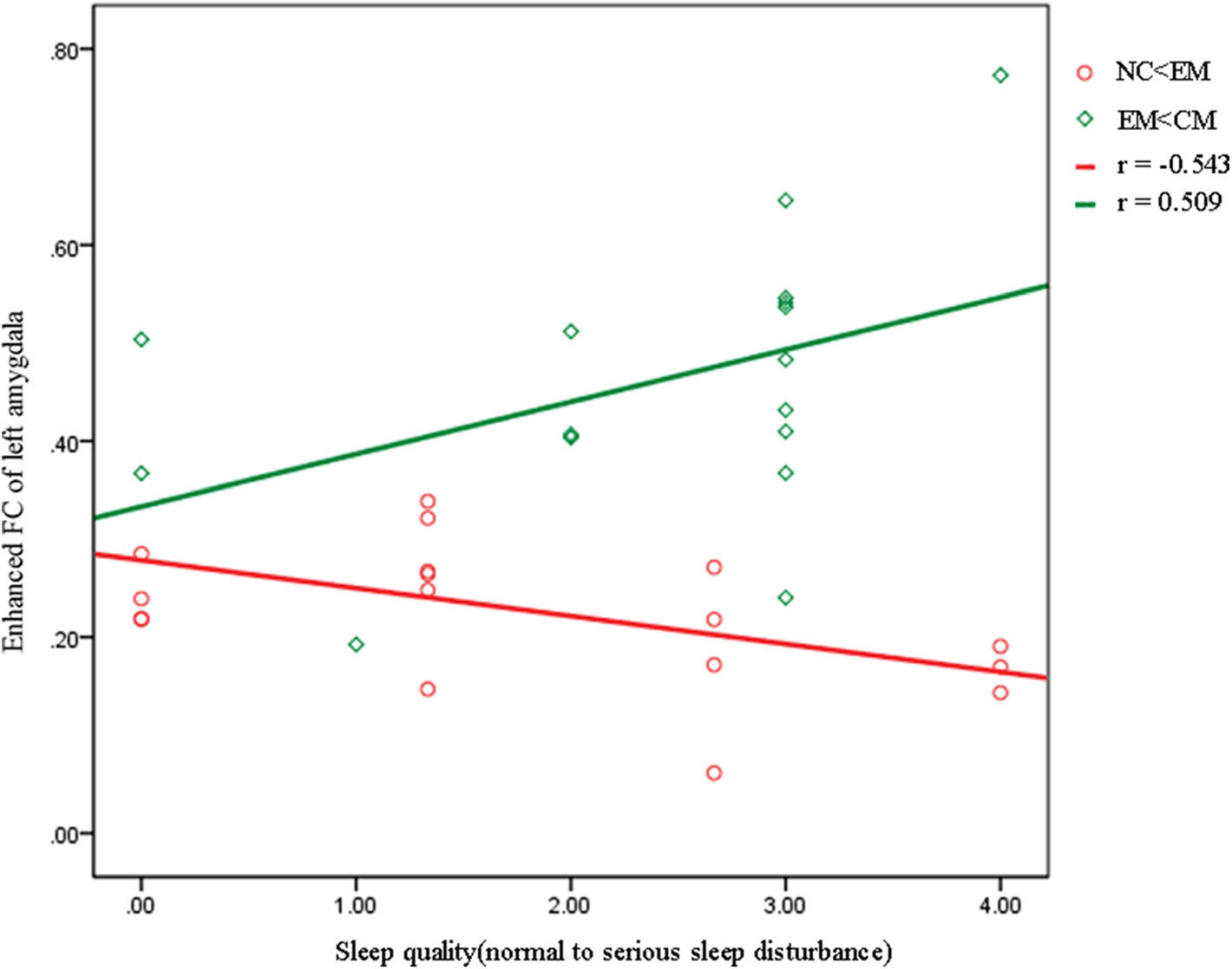
The correlation of the score of sleep quality with the enhanced FC of left amygdala in EM compared with NC (red circle and line) and in CM compared with EM (green rhombus and line). NC < EM, the increased FC of left amygdala in EM compared with NC; EM < CM, the increased FC of left amygdala in CM compared with EM

The correlation analysis showed a negative correlation between the score of sleep quality (0, normal; 1, mild sleep disturbance; 2, moderate sleep disturbance; 3, serious sleep disturbance) and the increased FC of left amygdala in EM compared with NC, and a positive correlation between the score of sleep quality and the increased FC of left amygdala in CM compared with EM (Fig. 3). There was no significant correlation between the score of sleep quality and the altered FC strength of amygdala in CM compared with NC.

VAS, HAMA, HAMD and MoCA showed no significant correlation with altered FC of amygdala in comparison groups.

## Discussion

The relationship between pain and limbic system always receives much concern. As an essential element of the limbic system, the amygdala is worth of further study in migraineurs.

Our data demonstrated that there was no significant difference among NC, EM and CM, which demonstrated that amygdala volume could not be regarded as a progressive biomarker in migraine. Although a previous study showed the decreased amygdala volume in CM compared with EM based on voxel-based morphometry [17], the different results may be associated with the heterogeneous migraineurs in the study. The correlation analysis showed that there was no significant correlation between amygdala volume and the neuropsychological scale score, VAS, disease duration, which also suggested that amygdala volume could not be used to monitor the migraine progression. But the increased volume of the amygdala can be seen in the neuropathic pain, either in the study of animal models [18] or human subjects [19]. This hints that additional mechanisms or different influence intensity were involved in the pain of migraine on amygdala.

In our study, the enhanced FC of the left amygdala was observed in EM compared with NC, and the other altered amygdala FC was not revealed, which confirmed that left amygdala may be associated with the genesis of EM, and the enhanced FC may also be relevant to understanding the EM associated with psychopathology. This laterality pattern may be associated with the different functions of bilateral amygdala, and the left amygdala was known to contribute to the brain’s reward system. Therefore, the laterality mechanism should be further investigation.

Figure 2 showed the enhanced functional connectivity of bilateral amygdala in CM compared with EM. Just as in the animal modes study of neuropathic pain, the time-dependency of changes in the activity of the amygdala were shown [20]. Our study showed a similar time-dependency tendency and gave a direct evidence of central sensitization. By the ground of this enhanced FC, the animal trial proved that sensitization of neurons in the central nucleus of the amygdala via the decreased GABAergic inhibition contributes to the development of neuropathic pain-related anxiety-like behaviors [21].

This study showed there were no significant changes for FC of the right amygdala in NC vs. EM and EM vs. CM. However, there was a significant decreased FC of right amygdala in CM compared with NC. These altered FC pattern demonstrated that left and right amygdala played different roles in the genesis of EM and CM. The enhanced FC of left amygdala could underlie the genesis of EM, and contribute in the CM chronicization. While the decreased FC of right amygdala also could underlie the genesis of CM, and some brain regions with increased FC of right amygdala may participate in CM chronicization based on the comparison of EM and CM. The altered FC pattern of left and right amygdala may be associated with different functions of bilateral amygdala. The left amygdala mainly induced pleasant or unpleasant emotions, while the right amygdala mainly induced negative emotions such as fear and sadness [22]. Therefore, left and right amygdala played the different roles in the genesis of EM and CM.

Based the Table 2, the brain regions with evident enhanced connectivity were inferior temporal gyrus (ITG) and orbitofrontal gyrus (OFG). ITG is located on the inferior convexity of the temporal lobe in human, and it participated in the analysis of visual form and object recognition, and is considered to be the final stage in the ventral cortical visual system [23]. Voxel-based morphometry demonstrated that gray matter volume decreased in right ITG in CM patients [24], and our study confirmed the enhanced connectivity between ITG and amygdala, which may confirmed that ITG played a crucial role in the neurolimbic pain-modulation network. A study of effects of long-term acupuncture treatment on resting-state brain activity in migraine patients revealed that a decrease in ReHo values was observed after treatment in the left ITG [25], which indicated that the resting activity of ITG could be used for the evaluation of the therapy of migraine, and the enhanced FC between ITG and amygdala may provide a new clue to reveal the neuromechanism of migraine.

Acute and chronic pain may involve multiple brain regions for the pain processing, and they are interconnected to construct the pain network [26]. Regarding migraine, the pain genesis may embrace the acute and chronic process, such as EM and CM. OFG, anatomically called as ventromedial prefrontal cortex, is participated the sensory integration [27] and the expected reward and punishment of an action, and it shares extensive reciprocal connections with the amygdala. Fumal et al. demonstrated that OFG was involved chronic migraine evolving from episodic migraine, and showed persistent orbitofrontal hypofunction after withdrawal of analgesics [28]. The disrupted OFG connectivity or circuitry could affect the decision-making, emotion regulation and reward expectation, however, our study demonstrated the enhanced connectivity was involved between the OFC and amygdala in CM evolving from EM. This connectivity pattern demonstrated that amygdala participated the central sensitization, and the mechanism should be worth further study.

Our data also showed the enhanced connectivity between left amygdala and left middle cingulate cortex (MCC) in EM compared with NC, and between the right amygdala and right anterior cingulate cortex (ACC) and MCC. ACC played a role in pain modulation, analgesia, and attention and anticipation of pain [26, 29]. Although a recent VBM study demonstrated that the decreased ACC grey matter was observed in CM compared with EM [17], our study showed enhanced connectivity of the amygdala with ACC in CM, and this indicated the amygdala played a key role in the pain modulation of ACC. A recent study revealed that MCC also showed pain-induced activation after painful heat stimulus in EM compared with NC [30], and Teutsch et al. demonstrated that increased grey matter was observed after noxious stimuli were applied repetitively to NC [31]. In our study, enhanced connectivity between amygdala and MCC was observed, and this connectivity pattern suggested the amygdala modulation network had a crucial role in the EM and CM genesis.

The relationship between migraine and sleep has been reported, and there was a significant association between severe sleep disturbance and primary headache disorder, especially in CM [32]. The insufficient sleep may induce a migraine attack, and lack of adequate rest might be an attack-precipitating and hyperalgesia-inducing factor [33–35]. A large clinical sample of migraineurs study with 1283 migraineurs revealed the substantial sleep/migraine relationship, and implicate sleep disturbance in specific headache patterns and severity [36]. However, the precise mechanism remained poorly understood up to now.

The amygdala plays a role in REM sleep modulation [37], and functional interactions between the amygdala and the cortex was demonstrated by PET data during REM sleep [38]. An animal study demonstrated that amygdala played a role in sleep regulation and sleep disturbance may be associated with psychopathology [39], and a recent resting-state functional MRI showed that altered amygdala FC was investigated after 36 h of total sleep deprivation [40]. Therefore, sleep disturbance may involve the functions of amygdala. In this study, an increased FC of left amygdala in EM compared with NC and in CM compared with EM. The correlation analysis demonstrated that there was a negative correlation between the degree of sleep disturbance and the increased FC of left amygdala in EM, which suggested that sleep disturbance may play a limited role in the EM attack. However, a positive correlation was investigated between the degree of sleep disturbance and the increased FC of left amygdala in CM compared with EM, which confirmed that sleep disturbance may play a key role in CM genesis evolved from EM. Therefore, these sleep/increased FC of left amygdala relationship could explained the fact that sleep complaints occurred with greater frequency among CM than EM [36]. Although the results were interesting to reveal the role of sleep quality by the enhanced amygdala FC in EM and CM genesis, it should be cautious to explain the relationship between the sleep disturbance and migraine genesis since the left amygdala could induced pleasant or unpleasant emotion [22].

The present study has some limitations. First, the number of EM and CM patients was relatively small, and a large sample study would be need in future study. Second, this study was a cross-sectional study, and the longitudinal observation should be performed to investigate the evolvement of EM to CM. Lastly, the effect of emotion regulation or lability on the change of amygdala FC in migraine would be investigated in the future because of well-known emotional correlates of amygdala [22, 41].

## Conclusions

The present study is the first to address the roles of amygdala in the neurolimbic pain-modulating in the migraine pathogenesis. Enhanced FC of the left amygdala in EM and decreased FC of the right amygdala in CM could elucidate the different neuromechanism of migraine. The enhanced FC of bilateral amygdala gave a direct evidence of central sensitization from EM to CM. And the sleep/increased FC of left amygdala relationship further enlightened the neuromechanism of enhanced FC of amygdala in neurolimbic pain network dysfunction. Potential treatment may be supported by the mechanism of this limbic system change.

## Abbreviations

CM: Chronic migraine
EM: Episodic migraine
NC: Normal control

## References

[1] Scher AI, Gudmundsson LS, Sigurdsson S, Ghambaryan A, Aspelund T, Eiriksdottir G (2009). Migraine headache in middle age and late-life brain infarcts. JAMA.

[2] Yu S, Liu R, Zhao G, Yang X, Qiao X, Feng J (2012). The prevalence and burden of primary headaches in China: a population-based door-to-door survey. Headache.

[3] Lipton RB (2009). Tracing transformation: chronic migraine classification, progression, and epidemiology. Neurology.

[4] Maizels M, Aurora S, Heinricher M (2012). Beyond neurovascular: migraine as a dysfunctional neurolimbic pain network. Headache.

[5] Hadjikhani N, Ward N, Boshyan J, Napadow V, Maeda Y, Truini A (2013). The missing link: enhanced functional connectivity between amygdala and visceroceptive cortex in migraine. Cephalalgia.

[6] Malick A, Burstein R (2000). Peripheral and central sensitization during migraine. Funct Neurol.

[7] Dodick D, Silberstein S (2006). Central sensitization theory of migraine: clinical implications. Headache.

[8] Curatolo M, Arendt-Nielsen L, Petersen-Felix S (2006). Central hypersensitivity in chronic pain: mechanisms and clinical implications. Phys Med Rehabil Clin N Am.

[9] Dehbandi S, Speckmann EJ, Pape HC, Gorji A (2008). Cortical spreading depression modulates synaptic transmission of the rat lateral amygdala. Eur J Neurosci.

[10] Mainero C, Boshyan J, Hadjikhani N (2011). Altered functional magnetic resonance imaging resting-state connectivity in periaqueductal gray networks in migraine. Ann Neurol.

[11] Akcali D, Sayin A, Sara Y, Bolay H (2010). Does single cortical spreading depression elicit pain behaviour in freely moving rats?. Cephalalgia.

[12] Headache Classification Committee of the International Headache Society (IHS) (2013) The International Classification of Headache Disorders, 3rd edition (beta version). Cephalalgia 33:629–808. 10.1177/0333102413485658 23771276

[13] Fischl B, Salat DH, Busa E, Albert M, Dieterich M, Haselgrove C (2002). Whole brain segmentation: automated labeling of neuroanatomical structures in the human brain. Neuron.

[14] Fischl B, Salat DH, van der Kouwe AJ, Makris N, Segonne F, Quinn BT, Dale AM (2004). Sequence-independent segmentation of magnetic resonance images. Neuroimage.

[15] Song XW, Dong ZY, Long XY, Li SF, Zuo XN, Zhu CZ (2011). REST: a toolkit for resting-state functional magnetic resonance imaging data processing. PLoS One.

[16] Tzourio-Mazoyer N, Landeau B, Papathanassiou D, Crivello F, Etard O, Delcroix N (2002). Automated anatomical labeling of activations in SPM using a macroscopic anatomical parcellation of the MNI MRI single-subject brain. Neuroimage.

[17] Valfre W, Rainero I, Bergui M, Pinessi L (2008). Voxel-based morphometry reveals gray matter abnormalities in migraine. Headache.

[18] Goncalves L, Silva R, Pinto-Ribeiro F, Pego JM, Bessa JM, Pertovaara A (2008). Neuropathic pain is associated with depressive behaviour and induces neuroplasticity in the amygdala of the rat. Exp Neurol.

[19] Mao C, Wei L, Zhang Q, Liao X, Yang X, Zhang M (2013). Differences in brain structure in patients with distinct sites of chronic pain: A voxel-based morphometric analysis. Neural Regen Res.

[20] Goncalves L, Dickenson AH (2012). Asymmetric time-dependent activation of right central amygdala neurones in rats with peripheral neuropathy and pregabalin modulation. Eur J Neurosci.

[21] Jiang H, Fang D, Kong LY, Jin ZR, Cai J, Kang XJ (2014). Sensitization of neurons in the central nucleus of the amygdala via the decreased GABAergic inhibition contributes to the development of neuropathic pain-related anxiety-like behaviors in rats. Mol Brain.

[22] Lanteaume L, Khalfa S, Regis J, Marquis P, Chauvel P, Bartolomei F (2007). Emotion induction after direct intracerebral stimulations of human amygdala. Cereb Cortex.

[23] Denys K, Vanduffel W, Fize D, Nelissen K, Peuskens H, Van Essen D, Orban GA (2004). The processing of visual shape in the cerebral cortex of human and nonhuman primates: a functional magnetic resonance imaging study. J Neurosci.

[24] Yu S, Chen X, Chen Z, Dong Z, Ma L (2014). EHMTI-0307. Chronification of migraine: a clinical and voxel-based morphometry study. J Headache Pain.

[25] Zhao L, Liu J, Zhang F, Dong X, Peng Y, Qin W (2014). Effects of Long-Term Acupuncture Treatment on Resting-State Brain Activity in Migraine Patients: A Randomized Controlled Trial on Active Acupoints and Inactive Acupoints. PLoS One.

[26] Peyron R, Laurent B, Garcia-Larrea L (2000). Functional imaging of brain responses to pain. A review and meta-analysis (2000). Neurophysiol Clin.

[27] Kringelbach ML (2005). The human orbitofrontal cortex: linking reward to hedonic experience. Nat Rev Neurosci.

[28] Fumal A, Laureys S, Di Clemente L, Boly M, Bohotin V, Vandenheede M (2006). Orbitofrontal cortex involvement in chronic analgesic-overuse headache evolving from episodic migraine. Brain.

[29] Tessitore A, Russo A, Esposito F, Giordano A, Taglialatela G, De Micco R (2011). Interictal cortical reorganization in episodic migraine without aura: an event-related fMRI study during parametric trigeminal nociceptive stimulation. Neurol Sci.

[30] Schwedt TJ, Chong CD, Chiang CC, Baxter L, Schlaggar BL, Dodick DW (2014). Enhanced pain-induced activity of pain-processing regions in a case-control study of episodic migraine. Cephalalgia.

[31] Teutsch S, Herken W, Bingel U, Schoell E, May A (2008). Changes in brain gray matter due to repetitive painful stimulation. Neuroimage.

[32] Odegard SS, Engstrom M, Sand T, Stovner LJ, Zwart JA, Hagen K (2010). Associations between sleep disturbance and primary headaches: the third Nord-Trondelag Health Study. J Headache Pain.

[33] Alstadhaug K, Salvesen R, Bekkelund S (2007). Insomnia and circadian variation of attacks in episodic migraine. Headache.

[34] Engstrom M, Hagen K, Bjork MH, Stovner LJ, Gravdahl GB, Stjern M, Sand T (2013). Sleep quality, arousal and pain thresholds in migraineurs: a blinded controlled polysomnographic study. J Headache Pain.

[35] Haque B, Rahman KM, Hoque A, Hasan AT, Chowdhury RN, Khan SU (2012). Precipitating and relieving factors of migraine versus tension type headache. BMC Neurol.

[36] Kelman L, Rains JC (2005). Headache and sleep: examination of sleep patterns and complaints in a large clinical sample of migraineurs. Headache.

[37] Siegel JM (2006). The stuff dreams are made of: anatomical substrates of REM sleep. Nat Neurosci.

[38] Maquet P, Franck G (1997). REM sleep and amygdala. Mol Psychiatry.

[39] Benca RM, Obermeyer WH, Shelton SE, Droster J, Kalin NH (2000). Effects of amygdala lesions on sleep in rhesus monkeys. Brain Res.

[40] Shao Y, Lei Y, Wang L, Zhai T, Jin X, Ni W (2014). Altered resting-state amygdala functional connectivity after 36 h of total sleep deprivation. PLoS One.

[41] Perlman G, Simmons AN, Wu J, Hahn KS, Tapert SF, Max JE (2012). Amygdala response and functional connectivity during emotion regulation: A study of 14 depressed adolescents. J Affect Disord.

